# The von Hippel-Lindau Chuvash mutation in mice alters cardiac substrate and high-energy phosphate metabolism

**DOI:** 10.1152/ajpheart.00912.2015

**Published:** 2016-07-15

**Authors:** Mary Slingo, Mark Cole, Carolyn Carr, Mary K. Curtis, Michael Dodd, Lucia Giles, Lisa C. Heather, Damian Tyler, Kieran Clarke, Peter A. Robbins

**Affiliations:** Department of Physiology, Anatomy, and Genetics, University of Oxford, Oxford, United Kingdom

**Keywords:** hypoxia-inducible factor, magnetic resonance imaging, hyperpolarized pyruvate

## Abstract

Hypoxia-inducible factor (HIF) appears to function as a global master regulator of cellular and systemic responses to hypoxia. HIF pathway manipulation is of therapeutic interest; however, global systemic upregulation of HIF may have as yet unknown effects on multiple processes. We used a mouse model of Chuvash polycythemia (CP), a rare genetic disorder that modestly increases expression of HIF target genes in normoxia, to understand what these effects might be within the heart. An integrated in and ex vivo approach was employed. Compared with wild-type controls, CP mice had evidence (using in vivo magnetic resonance imaging) of pulmonary hypertension, right ventricular hypertrophy, and increased left ventricular ejection fraction. Glycolytic flux (measured using [^3^H]glucose) in the isolated contracting perfused CP heart was 1.8-fold higher. Net lactate efflux was 1.5-fold higher. Furthermore, in vivo ^13^C-magnetic resonance spectroscopy (MRS) of hyperpolarized [^13^C_1_]pyruvate revealed a twofold increase in real-time flux through lactate dehydrogenase in the CP hearts and a 1.6-fold increase through pyruvate dehydrogenase. ^31^P-MRS of perfused CP hearts under increased workload (isoproterenol infusion) demonstrated increased depletion of phosphocreatine relative to ATP. Intriguingly, no changes in cardiac gene expression were detected. In summary, a modest systemic dysregulation of the HIF pathway resulted in clear alterations in cardiac metabolism and energetics. However, in contrast to studies generating high HIF levels within the heart, the CP mice showed neither the predicted changes in gene expression nor any degree of LV impairment. We conclude that the effects of manipulating HIF on the heart are dose dependent.

## Abstract (precis)

*This is the first integrative metabolic and functional study of the effects of modest hypoxia-inducible factor manipulation within the heart. Of particular note, the combination (and correlation) of perfused heart metabolic flux measurements with the new technique of real-time in vivo magnetic resonance spectroscopy using hyperpolarized pyruvate is a novel development*.

## NEW & NOTEWORTHY

*This is the first integrative metabolic and functional study of the effects of modest hypoxia-inducible factor manipulation within the heart. Of particular note, the combination (and correlation) of perfused heart metabolic flux measurements with the new technique of real-time in vivo magnetic resonance spectroscopy using hyperpolarized pyruvate is a novel development*.

prolonged hypoxia results in diverse changes within multiple organ systems: ventilatory acclimatization, increased erythropoiesis, pulmonary vascular remodeling, and metabolic alterations occur (54). Many of these diverse cellular and systemic responses to hypoxia are, or are likely to be, coordinated by the hypoxia-inducible factor (HIF) family of transcription factors. HIF is a heterodimer, comprising an oxygen-regulated α-subunit (HIF-1α, HIF-2α, or HIF-3α) and a ubiquitous β-subunit (HIF-1β). The stability of HIF-α subunits is regulated by oxygen-dependent prolyl hydroxylation [by prolyl-4-hydroxylase domain proteins (PHDs)], which enables recognition by the von Hippel-Lindau (VHL) ubiquitin E3 ligase and subsequent degradation via the ubiquitin-proteasome pathway (31, 54). Hence, HIF appears to function as a global master regulator of cellular and systemic responses to hypoxia in all metazoan species studied to date (36).

There is considerable interest in understanding further the role of HIF in both individual organ systems and within integrative physiology, driven in part by the potential benefits of VHL-HIF pathway manipulation in the treatment of cancer and vascular disease (49). For example, global systemic upregulation of HIF may have as yet unknown effects on multiple cellular and physiological processes. We sought to understand what these effects might be within the heart.

Exposure of animals and humans to sustained hypoxia results in a consistent pattern of myocardial alterations in metabolic gene expression, substrate utilization, energetics, and function. The dynamic changes in gene expression increase the capacity for glucose uptake (63) and utilization (1, 62), whereas capacity for fatty acid utilization is reduced (62). Indeed, myocardial glucose uptake is increased in high-altitude natives (21) and in rats exposed to hypobaric hypoxia (28). These alterations in substrate utilization may affect myocardial energetics and function: lower phosphocreatine (PCr)-to-ATP ratios were observed in Sherpa hearts (20), in lowlanders exposed to 20 h of normobaric hypoxia (with accompanying diastolic dysfunction) (22), and in trekkers travelling to Everest base camp at 5,300 meters (also with impaired diastolic function) (23).

It is of note that the cardiac metabolic changes seen in response to hypoxia are similar to those observed in heart failure (34, 43–45). Indeed, it has been hypothesized that myocardial hypoxia, with consequent activation of the HIF pathway, may play a role in altering cardiac substrate utilization, energetics, and contractile function; these changes may then cause or exacerbate heart failure. This hypothesis has been explored in experiments producing very high levels of HIF in the heart, from which it is clear that major alterations in HIF give rise to metabolic changes and cardiomyopathy (7, 24, 29, 35, 40, 41). However, these experiments do not reveal whether more modest alterations in HIF, particularly at a systemic level, produce similar results.

Chuvash polycythemia (CP) is a rare autosomal recessive disorder with the potential to address this question. CP arises from a single point mutation in *VHL*, which diminishes the binding affinity of the protein for hydroxylated HIF-1α and HIF-2α, increasing the expression of HIF target genes under normoxic conditions (2, 14). Patients with CP develop polycythemia, pulmonary hypertension, increased ventilatory and pulmonary vascular sensitivity to hypoxia, and altered skeletal muscle metabolism and energetics (14, 65) although any effects on cardiac function remain unidentified. Many aspects of the human disease have been shown to be faithfully recapitulated in the mouse model (18, 19, 39, 64).

The CP mouse therefore provides a unique opportunity to study the effects on the heart of long-term systemic activation of the HIF pathway at more “physiological” levels, without the confounding influence of periods of reduced oxygen availability through hypoxia. The purpose of the present study was to investigate the cardiac metabolic and functional phenotype of CP, using both in and ex vivo techniques.

## METHODS

### 

#### Animals.

CP breeding pairs were donated, and the original mutation was generated on a C57BL6 background as described previously (18). CP and wild-type (homozygous, WT) mice (from the same breeding colony of mice heterozygous for the *VHL* Chuvash mutation) were used for all comparisons. All animal procedures were compliant with both the United Kingdom Home Office Animals (Scientific Procedures) Act 1986, institution licence no. 30/2306, and the Local Ethical Review Procedures (University of Oxford Medical Sciences Division Ethical Review Committee). Mice were housed within individually ventilated cages, in room air, with free access to food and water.

#### Hematology and analysis of plasma metabolites.

Following excision of the heart for perfusion, blood was collected immediately from the chest cavity. Hematocrits, in a heparinized capillary tube, were measured using a hematocrit centrifuge (Hettich); hemoglobin was measured using a HemoCue Hb 201+ (HemoCue). The remaining blood was centrifuged at 4°C, and the plasma was subsequently frozen in liquid nitrogen. All plasma analysis was performed on an ABX Pentra 400 Clinical Chemistry analyzer (Horiba).

#### Cardiac magnetic resonance imaging.

Anesthesia was induced using 5%, and maintained with 1–2%, isoflurane in 100% O_2_. Respiratory rate and ECG were monitored continuously. Cine magnetic resonance imaging (MRI) was performed as described previously (58). Mice were placed in a purpose-built cradle that was lowered in a vertical-bore 11.7-T (500-MHz) system (Magnex) with a 40-mm birdcage coil (Bruker). A stack of contiguous 1-mm-thick true short-axis ECG- and respiration-gated cine/FLASH images were acquired to cover the entire heart. Image data were analyzed using the ImageJ software (NIH Image). End-systole (ES) and end-diastole (ED) frames for each slice were identified, and the left ventricle (LV) endo- and epicardial borders were outlined in all slices to give values for end-systolic volume (ESV) and end-diastolic volume (EDV). Stroke volume (SV = EDV − ESV), ejection fraction [= (SV/EDV) × 100], and cardiac output (= SV × heart rate) were calculated. LV mass was calculated as the product of the LV volume and the specific gravity of myocardium (1.05 g/cm^3^). The midventricular slice was defined as the one in which the papillary muscles were most prominent; right ventricle (RV) free wall thickness was measured in three separate locations in this slice, and subsequently averaged.

To quantify septal bowing (the distortion of the LV), a novel method was used. First, the LV epicardial border in early diastole in the midventricular slice was outlined, giving an actual perimeter length (*P*_A_) and area (*A*_A_). The maximum possible area enclosed by a perimeter of any given length arises when the figure is a circle. For a perimeter of length *P*_A_, we denote this area as *A*_C_. If the LV were perfectly circular, then the ratio of *A*_A_ to *A*_C_ would be 1. As the LV becomes increasingly distorted in shape *A*_A_ falls relative to *A*_C_, and hence the ratio will fall below 1. This “septal bowing ratio” thus enables quantification of the distortion of the septum using the LV itself as the comparator. We chose to quantify septal bowing in this way, since it is an approach that is likely to be relatively insensitive to observer variability. We expect that a relatively linear relationship would exist between this index and other indexes of septal bowing, such as the ratio between the long and short axes in a short-axis ventricular slice [as introduced by Ryan et al. (56) and which correlates with pulmonary artery pressure in patients with pulmonary hypertension], but this has not been tested directly.

#### Gene expression (real-time PCR).

Total RNA was extracted from 20–30 mg powdered (in liquid nitrogen) whole heart tissue (following Langendorff perfusion) and whole lung tissue. Total RNA was extracted using the Rneasy Fibrous Kit (Qiagen), including a DNase treatment step, and cDNA immediately synthesized from 1 μg RNA using the Applied Biosystems High Capacity cDNA Reverse Transcription Kit (Life Technologies). Real-Time PCR was performed using an ABI Prism 7000 Sequence Detection System (Applied Biosystems) with TaqMan Universal PCR Master Mix and TaqMan Gene Expression Assays (choosing manufacturer-recommended assays; Applied Biosystems). Relative quantification of mRNA expression levels was determined using the standard curve method and normalized to β-actin (heart tissue) or 18s ribosomal RNA (lung tissue).

#### Gene expression (microarray).

Total RNA was extracted from 20–30 mg powdered (in liquid nitrogen) whole heart tissue using the Rneasy Fibrous Kit (Qiagen), including a DNase treatment step. The RNA integrity was assessed on a BioAnalyzer (Agilent Laboratories); all samples had a RNA Integrity Number ≥7. Labeled sense single-strand DNA (ssDNA) for hybridization was generated from 200 ng starting RNA with the Ambion WT expression kit (P/N 4411973) and the Affymetrix GeneChip WT Terminal Labeling and Controls Kit (P/N 901525) according to the manufacturer's instructions. The distribution of fragmented sense ssDNA lengths was measured on the BioAnalyzer. The fragmented ssDNA was labeled and hybridized for 17 h at 45°C to the Affymetrix GeneChip Human Mouse 1.0 ST Array (Affymetrix). Chips were processed on an Affymetrix GeneChip Fluidics Station 450 and Scanner 3000. Affymetrix Command Console was used to generate cel files, and Affymetrix Expression Console was used for the quality control. Arrays were RMA normalized in GeneSpring GX 12, and differentially expressed genes were identified using Limma with a Benjamini and Hochberg multiple testing correction of ≤0.05. Because few genes were detected using this method, the data were reanalyzed using PLIER normalization and a Student's *t*-test with a *P* value cutoff of ≤0.05 and a fold change difference between WT and Chuvash of ≥1.3.

#### Isolated heart perfusion.

Mice were terminally anesthetized with an intraperitoneal injection of pentobarbitol sodium (500 mg/kg; Merial), and the heart was excised and arrested in ice-cold Krebs-Henseleit buffer; blood was immediately collected from the chest cavity for subsequent analysis. The ascending aorta was cannulated, and the heart was perfused retrograde in Langendorff mode at 37°C at a constant perfusion pressure of 80 mmHg. A polyethylene balloon, connected to a pressure transducer and used to measure cardiac function, was inserted in the LV lumen, and its volume was adjusted to produce an end-diastolic pressure of 4–8 mmHg. Rate pressure product (RPP) was calculated as the product of LV developed pressure (systolic pressure − end-diastolic pressure) and heart rate. Hearts were perfused with 100 ml of a modified Krebs-Henseleit recirculating buffer (in mmol/l: 118 NaCl, 3.7 KCl, 1.2 MgSO_4_, 1.97 CaCl_2_, 0.5 Na_2_EDTA, 25 NaHCO_3_, and 1.2 KH_2_PO_4_) containing 11 mmol/l glucose and 0.4 mmol/l palmitate prebound to 1.5% albumin as substrates. The buffer was continually gassed with a mix of 95% O_2_ with 5% CO_2_.

#### Perfused heart energetics.

Perfused hearts were inserted in an 11.7-T (500-MHz) vertical bore (123 mm internal diameter) magnet (Magnex Scientific) with a 10-mm probe (Rapid) containing concentric ^1^H- and ^31^P-sensitive coils. Fully relaxed ^31^P spectra were acquired using pulse-and-collect sequence at a repetition time of 10 s and a flip angle of 90° (32 averages, total acquisition time of 5 min, steady state). Approximate doubling of the RPP was then achieved by an infusion of isoproterenol (concentration received by the heart 2–5 nM), and further spectra were acquired (minimum acquisition time 5 min). Spectra were analyzed using the jMRUI software (42) to give values for PCr and γ-ATP abundance, and the ratio of these two. Spectra for each perfusion condition (standard, or isoproterenol) were averaged to give final values for PCr/ATP.

#### Measurement of cardiac substrate metabolism.

To determine glycolytic flux in the perfused heart, 50 μCi of d-[5-^3^H]glucose (Amersham) were added to the recirculating buffer. Following a 10-min stabilization period, perfusate samples were collected at 5-min intervals for 35 min. ^3^H_2_O was separated from the d-[5-^3^H]glucose in the time samples using Dowex ion exchange resin (Sigma) and subsequently used to calculate the glycolytic rate. Because of the position of the ^3^H label, this method gives a measure of true glycolytic rate since the label is cleaved by enolase in the cytosol.

Cardiac lactate efflux was determined by measuring lactate concentration spectrophotometrically in timed perfusate collections using lactate dehydrogenase. This method gives a measure of net lactate efflux from the heart. Cardiac palmitate oxidation rates were determined in a separate group of perfused hearts by adding 50 μCi of [9,10-^3^H]palmitate (Amersham) to the recirculating buffer and performing a chloroform-methanol Folch extraction on the time buffer samples to collect the ^3^H_2_O.

#### Measurement of in vivo cardiac metabolism in real time.

Mice were studied in the early absorptive (fed) state, between 1:00 and 11:00 A.M. They were anesthetized in isoflurane and O_2_, and intravenous access was gained using a 32-G tail-vein cannula. Mice were then positioned within a 7-T horizontal-bore MR scanner. Respiratory rate and ECG were monitored continuously. As described previously (12), 0.15 ml of hyperpolarized [^13^C_1_]pyruvate (0.48 mmol/kg) was injected over 10 s followed by a 0.05-ml flush of heparinized saline to clear the delivery line. Sixty individual ECG-gated ^13^C-MR cardiac spectra were acquired over 1 min following injection and subsequently analyzed using the AMARES algorithm in the jMRUI software package [version 4.0 (42)]. The peak areas of [^13^C_1_]pyruvate, [^13^C_1_]lactate, [^13^C_1_]alanine, and [^13^C_1_]bicarbonate at each time point were quantified and used as input data for a kinetic model, as described previously (12).

#### Statistical analysis.

Differences between groups were assessed using one-way analysis of variance using SPSS Statistics 19 (IBM).

## RESULTS

The purpose of this study was to determine the cardiac metabolic and functional phenotype of the CP mouse compared with the WT.

### 

#### General hematological and biochemical characteristics of the CP mouse model.

Body mass, basic hematology, and plasma metabolites are summarized in Table 1. Polycythemia in the CP mice was confirmed by demonstrating a modest increase in both hemoglobin and hematocrit, consistent with previous reports (18, 19, 64). No differences were seen in nonfasting plasma metabolites.

**Table 1.** Basic hematological and other parameters

	Wild Type	*n*	Chuvash	*n*	*P* Value
Body mass, g					
Male mice	35.9 ± 0.7	12	28.4 ± 0.4	12	< 0.001*
Female mice	25.3 ± 0.3	4	23.4 ± 0.5	8	0.016*
Hematocrit, %	43 ± 0.6	13	51 ± 1.0	13	< 0.001*
Hemoglobin, g/l	138 ± 3	16	158 ± 4	13	< 0.001*
Glucose, mmol/l	13.4 ± 1.0	7	13.4 ± 0.5	7	0.96
Hydroxybutyrate, mmol/l	0.18 ± 0.04	8	0.21 ± 0.06	7	0.65
Nonesterified fatty acid, mmol/l	0.15 ± 0.01	8	0.13 ± 0.02	7	0.33
Triacylglycerol, mmol/l	2.5 ± 0.2	7	2.2 ± 0.5	7	0.56
Cholesterol, mmol/l	1.7 ± 0.20	8	1.6 ± 0.05	7	0.44

Results are means ± SE; *n*, no. of mice. Hematological values, nonfasting plasma metabolites, and body masses of mice aged 6-7 mo are shown.

* *P* < 0.05.

#### CP mice have normal cardiac metabolic gene expression.

**Fig. 1.**
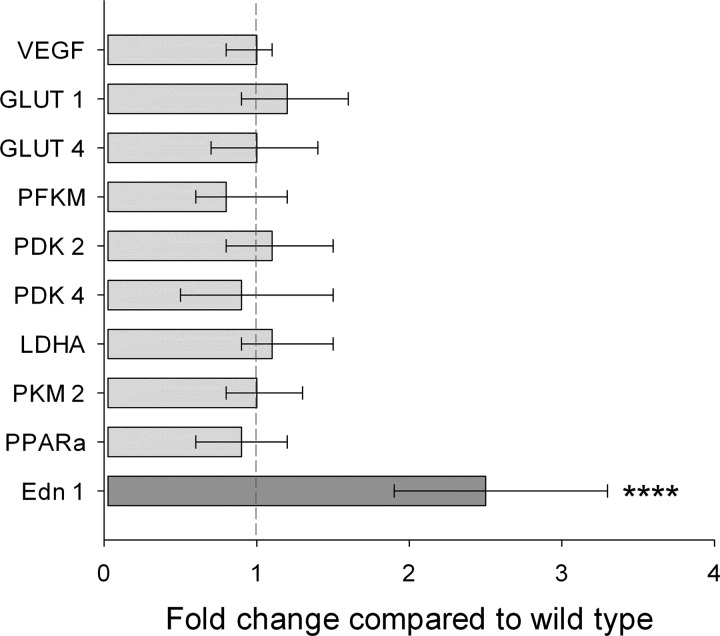
Cardiac and lung gene expression. Quantitative real-time PCR was used to measure expression of key metabolic genes in hearts from wild-type (WT, *n* = 9) and Chuvash polycythemia (CP, *n* = 7) mice, aged 6–7 mo. To confirm the methodology, expression of endothelin-1 (Edn 1; dark gray) was also determined in lungs from 13 WT and 11 CP mice. Values are means ± 95% confidence interval. VEGF, vascular endothelial growth factor; GLUT, glucose transporter; PFKM, phosphofructokinase; PDK, pyruvate dehydrogenase kinase; LDHA, lactate dehydrogenase A; PKM, pyruvate kinase muscle; PPARa, peroxisome proliferator-activated receptor α. ****P < 0.001.

The whole heart expression of key cardiac metabolic genes revealed no differences between WT and CP mice (Fig. 1). To confirm the methodology, the expression of lung endothelin-1 was studied and found to be significantly increased in the CP mice, in keeping with previous findings (19). The full microarray results are included in Supplemental Tables S1 and S2 (Supplemental material for this article is available online at the Journal website.).

#### CP mice exhibit features of pulmonary hypertension.

**Fig. 2.**
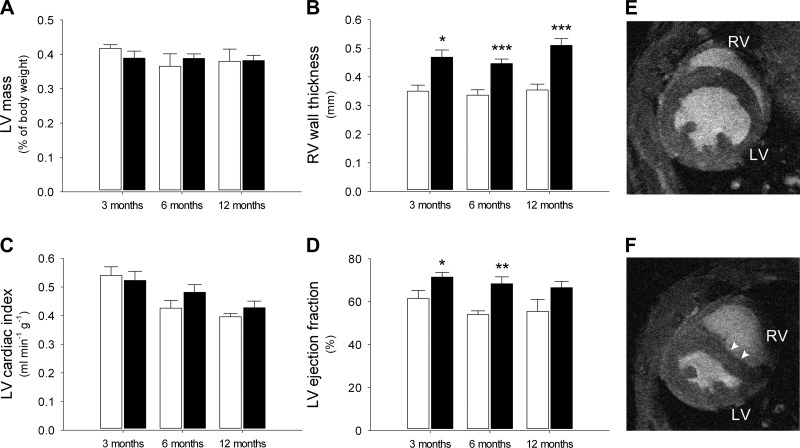
In vivo cardiac function. In vivo cine magnetic resonance imaging was used to measure cardiac mass and function in aging WT and CP mice (*n* = 3–8 mice/group). Open bars, WT; closed bars, CP. *A*: left ventricle (LV) mass; *B*: right ventricle (RV) wall thickness; *C*: LV cardiac output, corrected to body mass; *D*: LV ejection fraction. WT mice had normal cardiac morphology (*E*, representative image of mouse aged 3–6 mo). CP mice demonstrated marked interventricular septal bowing, particularly in early diastole (*F*; arrowheads, representative image of mouse aged 3–6 mo). *P < 0.05, **P < 0.02, and ***P < 0.01.

Cardiac function and mass in WT and CP mice at different ages are shown in Fig. 2. A full table of cine MRI results is included in Table 2.

**Table 2.** Full cine MRI data

	Wild Type	*n*	Chuvash	*n*	*P* Value
Body mass, g					
3 mo	28 ± 1.8	4	24 ± 1.3	7	0.093
6 mo	36 ± 1.1	4	25 ± 1.2	9	< 0.0001*
12 mo	37 ± 3.2	5	27 ± 0.9	8	0.003*
LV ejection fraction, %					
3 mo	61.5 ± 3.7	3	71.5 ± 2.2	6	0.043*
6 mo	54.0 ± 1.7	4	68.4 ± 3.2	8	0.014*
12 mo	55.5 ± 5.6	5	66.5 ± 2.9	8	0.078
LV mass, %body mass					
3 mo	0.42 ± 0.01	3	0.39 ± 0.02	6	0.262
6 mo	0.37 ± 0.04	5	0.39 ± 0.01	7	0.584
12 mo	0.38 ± 0.04	5	0.38 ± 0.02	8	0.952
MV RV wall thickness, mm					
3 mo	0.35 ± 0.02	3	0.47 ± 0.03	7	0.021*
6 mo	0.34 ± 0.02	5	0.45 ± 0.02	8	0.001*
12 mo	0.35 ± 0.02	5	0.51 ± 0.02	7	0.001*
Septal bowing ratio					
3 mo	0.91 ± 0.009	3	0.83 ± 0.03	7	0.038*
6 mo	0.92 ± 0.004	5	0.80 ± 0.01	7	< 0.0001*
12 mo	0.93 ± 0.0	4	0.83 ± 0.01	7	< 0.0001*
Heart rate, beats/min					
3 mo	420 ± 29	3	373 ± 8	6	0.069
6 mo	413 ± 10	5	398 ± 3	8	0.202
12 mo	418 ± 3	5	402 ± 7	8	0.129
Stroke volume, ml					
3 mo	35.1 ± 2.9	3	32.2 ± 1.2	7	0.282
6 mo	34.5 ± 1.0	5	30.8 ± 2.3	8	0.24
12 mo	37.1 ± 1.5	4	28.1 ± 1.4	8	0.003*
Cardiac output, ml/min					
3 mo	14.8 ± 2.0	3	12.5 ± 0.8	7	0.226
6 mo	14.3 ± 0.5	5	12.2 ± 0.9	8	0.116
12 mo	14.6 ± 1.2	5	11.3 ± 0.7	8	0.025*
LV cardiac index, ml·min^−1^·g^−1^					
3 mo	0.54 ± 0.03	3	0.52 ± 0.03	7	0.756
6 mo	0.43 ± 0.03	5	0.48 ± 0.03	8	0.197
12 mo	0.40 ± 0.01	5	0.43 ± 0.02	8	0.322

Results are means ± SE; *n*, no. of mice. For the "septal bowing ratio," the greater the distortion of the left ventricle (LV) by the septum, the greater the deviation from the perfect circle ratio of 1.0. MV, midventricular; RV, right ventricle.

* *P* < 0.05.

RV free wall hypertrophy, a typical feature of pulmonary hypertension, was seen in all age groups in the CP mice (Fig. 2*B*). These findings were comparable with those reported in a previous study in which pulmonary artery pressure and RV free wall thickness were measured using invasive techniques (19). In addition, marked interventricular septal bowing, another characteristic feature of pulmonary hypertension, was seen in all age groups in the CP mice (Fig. 2*F*). The degree of septal bowing was quantified and is included in Table 2.

In contrast to the marked changes in the RV, no differences were seen in LV mass or cardiac output (corrected for body mass; Fig. 2, *A* and *C*). Interestingly, LV ejection fraction was significantly increased in the younger CP mice, perhaps as a result of the pulmonary hypertension (Fig. 2*D*).

#### CP hearts exhibit more marked depletion of PCr under conditions of high workload.

**Fig. 3.**
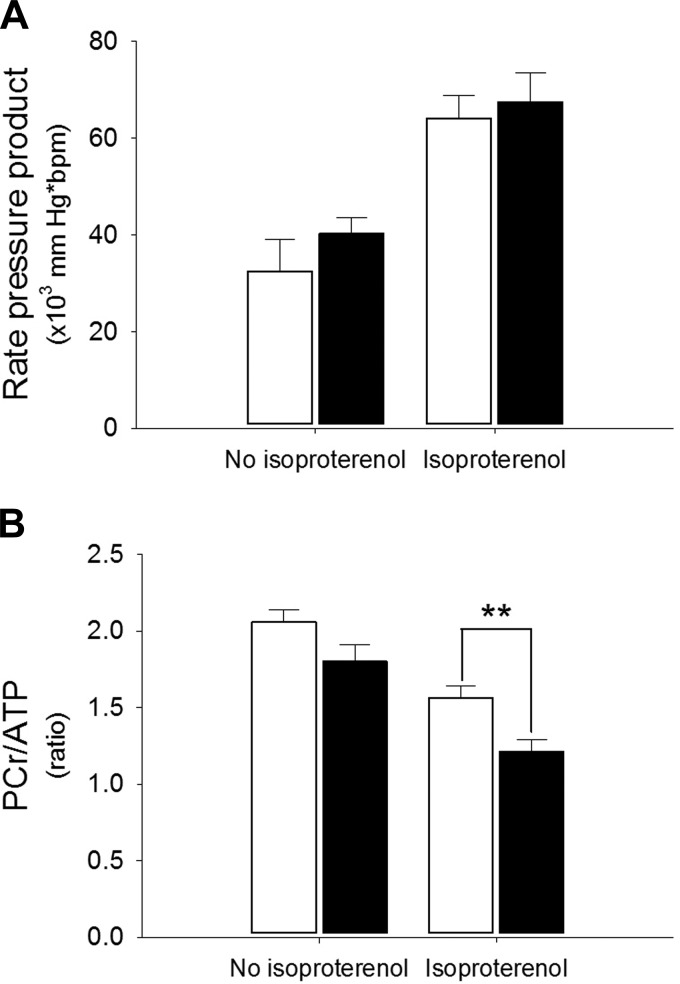
Perfused-heart energetics. ^31^P-magnetic resonance spectroscopy was performed on perfused hearts from WT and CP mice (*n* = 5 in each group), aged 6–7 mo. Body mass 30.1 ± 2.1 g for WT vs. 28.7 ± 1.2 for CP (not significant). Open bars, WT; closed bars, CP. Hearts were perfused under normal Langendorff conditions using palmitate buffer, and spectra were acquired, both without and with an infusion of isoproterenol to increase the rate pressure product (RPP). As expected, isoproterenol significantly increased the RPP (*P* < 0.001) and decreased the PCR-to-ATP ratio (*P* < 0.001). No significant difference between WT and CP was detected without isoproterenol, but following the increase in RPP the PCr/ATP ratio was significantly lower in the CP hearts compared with WT controls. *A*: RPP; *B*: PCr/ATP ratio. **P < 0.02.

^31^P-MR spectroscopy of Langendorff-perfused hearts enabled the measurement of cardiac energetics under conditions of both normal and high workload. Although cardiac energetics were similar in WT and CP hearts perfused under normal conditions, an infusion of isoproterenol (causing an approximate doubling in RPP) resulted in significantly lower PCr/ATP ratios in the CP hearts compared with WT controls (PCr/ATP 1.21 ± 0.08 in CP vs. 1.56 ± 0.08, *P* < 0.02) (Fig. 3).

#### Glycolytic flux and lactate efflux are increased in the CP heart.

**Fig. 4.**
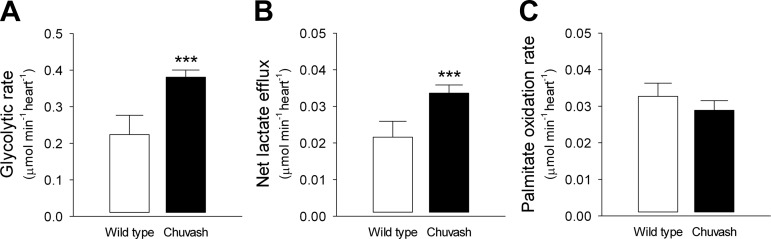
Perfused heart metabolism. Glycolytic flux (*A*) and net lactate efflux (*B*) were determined in WT (*n* = 4) and CP (*n* = 7) mice, aged 15–17 mo. Body mass 33.8 ± 1.8 g for WT vs. 29.4 ± 1.6 for CP (not significant). Glycolytic and lactate rates were highly correlated (Pearson *r* = 0.935, *P* < 0.001). Palmitate oxidation rates (*C*) were determined in separate mice (*n* = 5 in each group), aged 11–17 mo. Body mass 27.4 ± 1.5 g for WT vs. 26.2 ± 2.0 for CP (*P* = 0.034). ***P < 0.01.

Glycolytic flux in the perfused heart, measured using [^3^H]glucose, was 1.8-fold higher in the CP heart compared with WT controls (Fig. 4*A*). Analysis of variance, using genotype (CP or WT) as the fixed factor and heart mass and RPP as covariates, demonstrated that the CP mutation was the only significant determining factor on glycolytic rate (*P* = 0.004 for genotype; *P* = 0.095 for heart weight; *P* = 0.147 for RPP). Similarly, net lactate efflux was 1.5-fold higher in the CP hearts (*P* = 0.009 for genotype; *P* = 0.121 for heart weight; *P* = 0.123 for RPP) (Fig. 4*B*). There was a significant correlation between glycolytic and lactate efflux rates (Pearson *r* = 0.935, *P* < 0.0001). In contrast to the increased glucose utilization and lactate production by the CP hearts, a decrease in fatty acid oxidation [as might be predicted by the Randle cycle (27)] was undetectable (Fig. 4*C*).

#### In vivo pyruvate metabolism, measured in real time, is significantly altered in the CP heart.

**Fig. 5.**
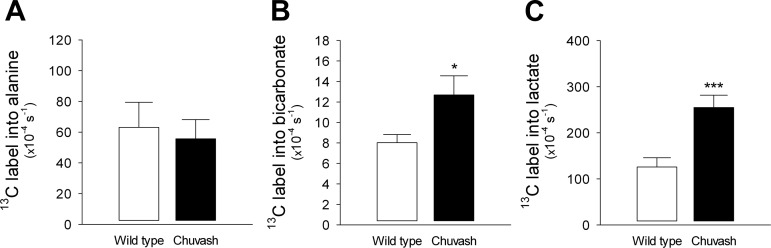
In vivo real-time cardiac metabolism. In vivo magnetic resonance spectroscopy of hyperpolarized [^13^C_1_]pyruvate was performed in WT (*n* = 5) and CP (*n* = 4) mice, aged 9–15 mo. Body mass 32.0 ± 3.0 g for WT vs. 27.0 ± 3.0 for CP (not significant). This technique allowed real-time measurement of the rate of label incorporation from pyruvate into alanine (*A*), bicarbonate (*B*), or lactate (*C*). *P < 0.05 and ***P < 0.01.

The advent of dynamic nuclear polarization (DNP) has enabled the study of cardiac metabolism in real time (59). In vivo ^13^C-MR spectroscopy of hyperpolarized [^13^C_1_]pyruvate revealed, in keeping with the perfusion studies, a twofold increase in the rate of ^13^C_1_ label incorporation in lactate in the CP hearts (*P* < 0.01, Fig. 5). Furthermore, there was a 1.6-fold increase in the rate of label incorporation in bicarbonate (*P* < 0.05), indicating elevated flux through pyruvate dehydrogenase (Fig. 5).

## DISCUSSION

It is now known that HIF functions as a global master regulator, coordinating diverse cellular and systemic responses to hypoxia. Studies on patients and mice have demonstrated that perturbations in the HIF pathway, such as CP or HIF-2α gain-of-function mutations, result in profound abnormalities in systemic and cellular processes that are usually under tight control. Marked changes in skeletal muscle metabolism, ventilation, pulmonary vascular tone, and glucose homeostasis are all observed in both humans and animals with disorders of the HIF pathway (2, 13–16, 18, 19, 37, 39, 46, 48, 50–53, 57, 64–66, 68, 69). We now demonstrate that chronic modest upregulation of HIF due to the Chuvash *VHL* mutation results in altered cardiac metabolism and energetics in the mouse heart.

A primordial function of HIF-1 is to find the optimal balance between oxidative and glycolytic metabolism for a given local oxygen concentration. As such, HIF-1 target genes, including glucose transporters and glycolytic enzymes (30, 60), lactate dehydrogenase (61) and pyruvate dehydrogenase kinase-1 (33, 47), enable increased glucose metabolism. In both hypoxia and hypoxia-mimetic models, myocardial expression of the master regulator of fatty acid metabolism, peroxisome proliferator-activated receptor-α (PPARα), is decreased together with its downstream targets such as pyruvate dehydrogenase kinase-4 (1, 55, 62). Alterations in cardiac mitochondrial oxidative metabolism are also seen (17). Thus, one would predict that exposure to hypoxia, or chronic activation of the HIF pathway, would result in an increased reliance upon glycolysis and glucose oxidation and a shift away from fatty acid β-oxidation.

Most studies investigating the role of HIF in cardiac metabolism and function have employed tactics either to overexpress HIF itself or to impair its degradation by using altered levels of PHDs or VHL. Mice with cardiomyocyte-specific loss of VHL have elevated HIF levels in the heart, accompanied by increased expression of glycolytic genes, cellular lipid accumulation, and progressive heart failure (35). Combined cardiac-specific loss of both PHD2 and PHD3 results in increased expression of phosphoglycerate kinase, decreased expression of PPARα, myocyte accumulation of lipid, and severe cardiomyopathy (41). Conditional somatic inactivation of PHDs produces similar results (40). Inducible cardiac-specific overexpression of an oxygen-stable form of HIF-1α results in increased transcript levels of glycolytic genes and progressively impaired cardiac function (7). Furthermore, long-term constitutive overexpression of HIF-1α results in increased glucose uptake and the development of cardiomyopathy (24). Taken together, these studies have shown that very high levels of HIF in the heart result in a switch in gene expression toward increased glucose metabolism, with accompanying contractile dysfunction. In contrast, shorter-term constitutive overexpression of HIF-1α mRNA in cardiomyocytes did not result in cardiomyopathy, but this construct only produced a barely detectable increase in HIF in normoxia. Interestingly, this overexpression was protective during myocardial infarction, where hypoxia would be expected to be present (32).

Our study investigated the cardiac effects of the Chuvash *VHL* mutation in mice, which results in systemic long-term modest upregulation of HIF. Our model faithfully recapitulated findings seen in previous studies on CP patients and mice [raised hemoglobin and hematocrit, pulmonary hypertension (shown using noninvasive MRI), and RV hypertrophy (using MRI, rather than histology)]. However, a surprising feature of our study was the apparent lack of change in the expression of key metabolic genes within the heart, despite clear alterations in substrate metabolism and energetics. It is possible that our whole heart analysis masked differential changes that could have been seen in the left, vs. the right, ventricle (1, 62). Alternatively, the presence of pulmonary hypertension and right ventricular hypertrophy may have resulted in genetic remodeling in addition to that caused by the Chuvash *VHL* mutation. Finally, it is possible that the LV in the CP mouse is exposed to a chronic increase in local and/or systemic sympathetic drive (9, 10, 38, 67), which may also alter gene transcription on the background of that caused by the HIF upregulation itself.

Previous studies of HIF manipulation within the mouse heart have demonstrated an increased reliance upon glucose metabolism, with accompanying contractile dysfunction (7, 24, 35, 40, 41). In the much more modest perturbation of the HIF system in the current study, these two features were not coupled, in keeping with a previous study demonstrating that short-term HIF mRNA overexpression could be cardioprotective (32). While there was clear evidence of increased glucose utilization and altered energetics, LV function was enhanced rather than diminished. It is likely that this increased ejection fraction is a reflection of the pulmonary hypertension demonstrated in the CP mice. One potential mechanism is that bowing of the interventricular septum arises as a consequence of the elevated pulmonary arterial pressure, and this could result directly in a reduced LV end-systolic volume. Another possible mechanism is that there is an overall increased sympathetic drive to the heart to maintain the cardiac output in the face of an elevated pulmonary vascular resistance. Again, this would be expected to result in a reduced LV end-systolic volume. It should also be noted that cell types other than cardiomyocytes may be relevant. For example, endothelial cell-specific HIF has been shown to influence cardiac glucose uptake, and this would have effects both in vivo and on the isolated perfused-heart preparation (26).

The balance between myocardial glucose and fatty acid metabolism can be studied using the combination of radiolabeled substrates and the isolated perfused heart (6, 8). We have demonstrated that the hearts from CP mice have elevated glycolytic flux, accompanied by increased net lactate efflux. This finding is in keeping with previous genetic studies that have shown that long-term constitutive overexpression of HIF-1α in the heart results in increased glucose uptake (24) and that cardiac-specific loss of HIF-1α causes decreased cardiomyocyte lactate concentrations (25). In our study, the increase in glycolytic flux was greater than that of pyruvate dehydrogenase (PDH) flux, meaning that the decrease in palmitate oxidation did not reach significance. However, following hypoxia, mouse hearts have been shown to have increased glycolytic flux, increased lactate production, and significantly decreased fatty acid oxidation (11).

The advent of DNP enables flux through metabolic pathways to be measured in real time in vivo. This technique has recently been optimized for use in small animals, such as mice, allowing genetic models to be studied for the first time (5, 12). The increased sensitivity [>10,000-fold (3)] of DNP allows the acquisition of data within 1 min after infusion of a bolus of hyperpolarized [^13^C_1_]pyruvate. This bolus is required to be relatively large; however, during the short time frame of data acquisition, no perturbation of plasma metabolites (other than pyruvate) or PDH activity occurs (4, 5). Using hyperpolarized [^13^C_1_]pyruvate MR spectroscopy, we have shown that there is increased rate of label incorporation from pyruvate in both lactate (via lactate dehydrogenase) and bicarbonate (via PDH). By using a combination of techniques, we have therefore demonstrated that the measurements of metabolic flux obtained ex vivo are in agreement with those made in real time in vivo.

Metabolic flexibility is required for the heart to continue to perform under conditions of varying workload. Inflexibility, due to increased reliance on a substrate such as glucose (which yields less ATP per molecule compared with fatty acids), may result in myocardial energy depletion. In agreement with this hypothesis, we have demonstrated that myocardial energetics, quantified by measurement of PCr and ATP, are reduced in the CP perfused heart under conditions of increased workload. Altered cardiac energetics are a feature of both heart failure (45) and hypoxia (20, 22, 23); our study adds to this body of evidence by demonstrating that modest long-term upregulation of HIF may result in a similar finding.

In summary, a modest systemic dysregulation of the HIF pathway, caused by the Chuvash *VHL* mutation, resulted in clear alterations in cardiac metabolism and energetics. However, in contrast to studies generating high levels of HIF in the heart, the CP mice showed neither the predicted changes in gene expression nor any degree of LV impairment. We conclude that the effects of manipulating HIF on the heart are dose dependent. In the present study, the pulmonary hypertension associated with CP clearly had a significant effect on cardiac function. In future studies employing the CP mutation to study modest HIF dysregulation, targeting this mutation specifically to cardiac myocytes could obviate the influence of pulmonary hypertension.

## GRANTS

This work was supported by the Wellcome Trust (WT090123AIA, to M. Slingo), Diabetes UK (11/0004175 to L. C. Heather), and by the British Heart Foundation (FS/10/002/28078 to D. Tyler). Equipment support was provided to D. Tyler by GE Healthcare, UK.

## DISCLOSURES

No conflicts of interest, financial or otherwise, are declared by the authors.

## AUTHOR CONTRIBUTIONS

M.S., M.A.C., L.C.H., D.J.T., K.C., and P.A.R. conception and design of research; M.S., M.A.C., C.A.C., M.K.C., M.S.D., L.G., and L.C.H. performed experiments; M.S., M.A.C., C.A.C., M.K.C., M.S.D., L.C.H., D.J.T., and K.C. analyzed data; M.S., M.A.C., C.A.C., M.K.C., M.S.D., L.C.H., D.J.T., K.C., and P.A.R. interpreted results of experiments; M.S. prepared figures; M.S. drafted manuscript; M.S., L.C.H., K.C., and P.A.R. edited and revised manuscript; M.S., M.A.C., C.A.C., M.K.C., M.S.D., L.G., L.C.H., D.J.T., K.C., and P.A.R. approved final version of manuscript.

## Supplementary Material

Supplemental Table
