# Supplementary material for: The von Hippel-Lindau Chuvash mutation in mice alters cardiac substrate and high-energy phosphate metabolism
Source: Am J Physiol Heart Circ Physiol. 2016 Jul 15;311(3):H759–67. doi: 10.1152/ajpheart.00912.2015 (PMC5142182; doi:10.1152/ajpheart.00912.2015)
Supplement: Supplemental Table [file Supplemental_Table.docx]

**The von Hippel-Lindau Chuvash mutation in mice alters cardiac substrate and high energy phosphate metabolism**

Mary Slingo, Mark Cole, Carolyn Carr, Mary K. Curtis, Michael Dodd, Lucia Giles, Lisa C Heather, Damian Tyler, Kieran Clarke, Peter A Robbins.

**Supplemental Table 2**

| Gene name | Fold change | Direction | *p* value |
| --- | --- | --- | --- |
| synuclein, alpha | 3.4 | up | 0.05 |
| alpha-2-macroglobulin | 3.1 | up | 4.9E-05 |
| myosin, light polypeptide 7, regulatory | 2.8 | down | 0.004 |
| myosin, light polypeptide 4 | 2.5 | down | 0.004 |
| sarcolipin | 2.0 | down | 0.03 |
| nuclear receptor subfamily 4, group A, member 3 | 2.0 | down | 0.04 |
| myosin binding protein H-like /// predicted gene 12522 | 1.9 | down | 0.02 |
| myosin, light polypeptide 1 | 1.9 | up | 7.9E-04 |
| nebulin | 1.8 | up | 1.0E-04 |
| arachidonate 5-lipoxygenase | 1.8 | down | 1.8E-04 |
| nephrocan | 1.7 | up | 0.02 |
| EF hand domain containing 1 | 1.7 | up | 0.002 |
| serine (or cysteine) peptidase inhibitor, clade A, member 1E/C/A | 1.6 | up | 1.1E-04 |
| dopachrome tautomerase | 1.6 | up | 0.007 |
| --- | 1.5 | up | 0.03 |
| interferon activated gene 205 | 1.5 | down | 0.03 |
| serine (or cysteine) peptidase inhibitor, clade A, member 1C /// serine (or cysteine) peptidase inhibitor, clade A, member 1A | 1.5 | up | 1.8E-04 |
| SH3-domain GRB2-like 2 | 1.5 | up | 0.003 |
| --- | 1.5 | up | 0.002 |
| --- | 1.5 | up | 0.04 |
| predicted gene 13152 /// predicted gene 13212 /// predicted gene 13154 | 1.5 | up | 0.03 |
| --- | 1.5 | up | 0.01 |
| chemokine (C-C motif) ligand 11 | 1.5 | down | 0.02 |
| centromere protein F | 1.5 | up | 0.03 |
| --- | 1.4 | up | 0.02 |
| solute carrier family 24 (sodium/potassium/calcium exchanger), member 2 | 1.4 | up | 0.03 |
| indolethylamine N-methyltransferase | 1.4 | down | 0.006 |
| --- | 1.4 | up | 0.04 |
| neuropeptide Y receptor Y1 | 1.4 | down | 0.006 |
| --- | 1.4 | up | 0.02 |
| --- | 1.4 | up | 0.004 |
| zinc finger, CCHC domain containing 5 | 1.4 | up | 0.008 |
| ubiquitin carboxy-terminal hydrolase L1 | 1.4 | up | 0.006 |
| lectin, galactose binding, soluble 6 /// lectin, galactose binding, soluble 4 | 1.4 | down | 0.007 |
| lectin, galactoside-binding, soluble, 3 binding protein | 1.4 | down | 0.04 |
| insulin-like growth factor binding protein 3 | 1.4 | down | 0.01 |
| actin, alpha 2, smooth muscle, aorta | 1.4 | up | 0.02 |
| plasmalemma vesicle associated protein | 1.4 | down | 7.5E-04 |
| cell death-inducing DNA fragmentation factor, alpha subunit-like effector B | 1.4 | up | 0.008 |
| expressed sequence AI593442 | 1.4 | up | 0.04 |
| dipeptidylpeptidase 6 | 1.4 | up | 0.01 |
| predicted gene 13154 | 1.4 | up | 0.006 |
| aspartate-beta-hydroxylase /// clavesin 1 | 1.4 | up | 0.02 |
| apolipoprotein B mRNA editing enzyme, catalytic polypeptide 1 | 1.4 | down | 0.05 |
| XIAP associated factor 1 | 1.4 | down | 0.05 |
| cystatin A | 1.4 | up | 0.009 |
| ganglioside-induced differentiation-associated-protein 10 | 1.4 | down | 0.02 |
| cadherin 11 | 1.4 | down | 0.003 |
| mucin 16 | 1.4 | down | 0.03 |
| * | 1.4 | down | 0.01 |
| * | 1.4 | down | 0.01 |
| * | 1.4 | down | 0.01 |
| * | 1.4 | down | 0.01 |
| CUB and zona pellucida-like domains 1 | 1.3 | up | 0.008 |
| --- | 1.3 | up | 0.03 |
| cysteine dioxygenase 1, cytosolic | 1.3 | up | 0.04 |
| aquaporin 4 | 1.3 | up | 0.01 |
| 6-phosphofructo-2-kinase/fructose-2,6-biphosphatase 1 | 1.3 | down | 0.02 |
| CUB and Sushi multiple domains 3 | 1.3 | up | 0.009 |
| G protein-coupled receptor 126 | 1.3 | down | 0.02 |
| --- | 1.3 | up | 0.02 |
| --- | 1.3 | up | 0.05 |
| --- | 1.3 | up | 0.008 |
| --- | 1.3 | up | 0.004 |
| --- | 1.3 | up | 0.009 |
| predicted gene 1078 | 1.3 | down | 0.05 |
| solute carrier family 12, member 5 | 1.3 | up | 0.002 |
| immunoglobulin superfamily, member 1 | 1.3 | up | 0.005 |
| beta-site APP-cleaving enzyme 2 | 1.3 | down | 0.001 |
| RIKEN cDNA 4930534B04 gene | 1.3 | up | 0.01 |
| --- | 1.3 | up | 0.02 |
| receptor (calcitonin) activity modifying protein 1 | 1.3 | down | 0.002 |
| slit homolog 2 (Drosophila) | 1.3 | down | 0.01 |
| PHD finger protein 11 | 1.3 | down | 0.01 |
| a disintegrin and metallopeptidase domain 34 /// predicted gene 5347 | 1.3 | up | 0.005 |
| regulator of G-protein signaling 5 | 1.3 | up | 0.01 |
| integrin alpha L | 1.3 | down | 0.05 |
| UBX domain protein 10 /// phospholipase A2, group IIC | 1.3 | up | 0.006 |
| transmembrane protein 196 | 1.3 | up | 0.04 |
| fibrinogen-like protein 2 | 1.3 | up | 0.02 |
| pleckstrin homology domain containing, family H (with MyTH4 domain) member 1 | 1.3 | up | 0.003 |
| KAT8 regulatory NSL complex subunit 1 | 1.3 | down | 0.04 |
| sodium channel, voltage-gated, type IX, alpha /// predicted gene 13629 | 1.3 | up | 0.04 |
| aldehyde dehydrogenase 1 family, member L1 | 1.3 | up | 0.003 |
| gap junction protein, alpha 5 | 1.3 | down | 0.002 |
| nephroblastoma overexpressed gene | 1.3 | down | 0.02 |
| dickkopf homolog 3 (Xenopus laevis) | 1.3 | down | 0.04 |
| --- | 1.3 | up | 0.03 |
| PDZ domain containing RING finger 4 | 1.3 | up | 0.02 |
| acyl-coenzyme A amino acid N-acyltransferase 2 | 1.3 | up | 0.02 |
| --- | 1.3 | down | 0.02 |

***Supplemental Table 2.* Full micro-array data.** Analysis performed using Plier normalisation and unpaired T test to compare groups. (* = chemokine (C-C motif) ligand 21A (serine) /// predicted gene 1987 /// chemokine (C-C motif) ligand 21C (leucine) /// c-C motif chemokine 21c-like /// chemokine (C-C motif) ligand 21B (leucine) /// predicted gene 10591 /// predicted gene 13304 /// c-C motif chemokine 21c-like)
